# Synthesis of two-dimensional Tl_*x*_Bi_1−*x*_ compounds and Archimedean encoding of their atomic structure

**DOI:** 10.1038/srep19446

**Published:** 2016-01-19

**Authors:** Dimitry V. Gruznev, Leonid V. Bondarenko, Andrey V. Matetskiy, Alexey N. Mihalyuk, Alexandra Y. Tupchaya, Oleg A. Utas, Sergey V. Eremeev, Cheng-Rong Hsing, Jyh-Pin Chou, Ching-Ming Wei, Andrey V. Zotov, Alexander A. Saranin

**Affiliations:** 1 Institute of Automation and Control Processes FEB RAS, 690041 Vladivostok, Russia; 2 Far Eastern Federal University, School of Natural Sciences, 690950 Vladivostok, Russia; 3 Institute of Strength Physics and Materials Science SB RAS, 634021 Tomsk, Russia; 4 Tomsk State University, 634050 Tomsk, Russia; 5 Institute of Atomic and Molecular Sciences, Academia Sinica, P.O. Box 23-166 Taipei, Taiwan; 6 Institute for Solid State Physics and Optics, Wigner Research Centre for Physics, Hungarian Academy of Sciences, Budapest, POB 49, H-1525, Hungary; 7 Department of Electronics, Vladivostok State University of Economics and Service, 690600 Vladivostok, Russia

## Abstract

Crystalline atomic layers on solid surfaces are composed of a single building block, unit cell, that is copied and stacked together to form the entire two-dimensional crystal structure. However, it appears that this is not an unique possibility. We report here on synthesis and characterization of the one-atomic-layer-thick Tl_*x*_Bi_1−*x*_ compounds which display quite a different arrangement. It represents a quasi-periodic tiling structures that are built by a set of tiling elements as building blocks. Though the layer is lacking strict periodicity, it shows up as an ideally-packed tiling of basic elements without any skips or halting. The two-dimensional Tl_*x*_Bi_1−*x*_ compounds were formed by depositing Bi onto the Tl-covered Si(111) surface where Bi atoms substitute appropriate amount of Tl atoms. Atomic structure of each tiling element as well as arrangement of Tl_*x*_Bi_1−*x*_ compounds were established in a detail. Electronic properties and spin texture of the selected compounds having periodic structures were characterized. The shown example demonstrates possibility for the formation of the exotic low-dimensional materials via unusual growth mechanisms.

The III-V compound semiconductor family possesses a wide range of properties from a wide band gap (5.96 eV) insulator BN to a smallest band gap (0.17 eV) semiconductor InSb with the highest mobility. As such, III-V materials are widely used in modern micro- and opto-electronics. The TlBi is not listed as a III-V semiconductor; it is expected to be metallic, supposedly can be stabilized in the tetragonal PbO^1^,^2^ or CaCl^3^ structure, but it seems that it has not been synthesized yet and does not exist in a bulk form^2^. However, Tl and Bi form a number of a bulk intermetallic material such as hexagonal BiTl_3_, cubic Bi_5_Tl_3_ and high-pressure Bi_2_Tl^4^ which have been studied for a long time starting from the pioneer work of Chikashige published as early as in 1906^5^. Remarkable that Tl-Bi compounds are superconductors in a wide compositional range with a critical temperature increases up to 5.4 K at 35 at.% Bi in the fcc phase^6^ though Bi is known not to be a superconductor at normal conditions.

Recently an interest to the low-dimensional III-V structures has risen. Following the lead of group-IV element low-dimensional nanostructures (graphene, in particular), planar sheets of II-VI (ZnO) and III-V (BN) materials attracted the attention. Calculations demonstrate the possible existence of 2D honeycomb structures (planar or buckled) of virtually each group III-V elements^7^,^8^, including TlBi^9^. Besides, the recent study has predicted that hypothetical GaBi, InBi and TlBi layers are large-gap 2D topological insulators^9^. These studies consider III-V sheets as single buckled honeycomb structures with equal amount of group III and V elements. Although it is close to a bilayer that can be found inside the bulk material, the real surface of III-V compound (which can be thought as a half-way to an isolated 2D sheet) demonstrates a 2 × 2 reconstruction with either one of the four group-III atoms missing (III-terminated surface) or three extra group-V atoms per unit cell (V-terminated surface)^10^. Additional prospect associated with hypothetical Tl-Bi 2D compound stems from the fact that both Tl and Bi are heavy metals with strong spin-orbit coupling and their monolayers on Si(111) or Ge(111)^11^,^12^,^13^,^14^,^15^,^16^,^17^,^18^,^19^,^20^ as well as their 2D compounds with other elements, e.g., Bi-Na, Tl-Pb^21^ and Tl-Sn^22^ on Si(111), demonstrate a giant Rashba-type spin-splitting of the surface-state bands, that makes them promising spintronic materials. In addition, exotic superconducting properties of the bulk Tl-Bi compounds provide a promise that Tl-Bi 2D compound could extend the list of recently found one-atomic-layer-thick superconductors^23^,^24^,^25^.

In the present study, the two-dimensional one-atomic-layer-thick Tl_*x*_Bi_1−*x*_ compounds (with *x* varying from 0.632 to 0.75) were formed by substituting appropriate amount of Tl in the monoatomic layer of Tl on Si(111) for Bi atoms. It appears that atomic arrangement of the forming Tl_*x*_Bi_1−*x*_ compounds is in variance with the honeycomb-like structures predicted theoretically. In contrast, they display a very unusual quasi-periodic ideally-packed tiling of a set of atomic-scale elements.

## Results

### Growth mode and tiling-like visualization of STM images

**Figure 1:**
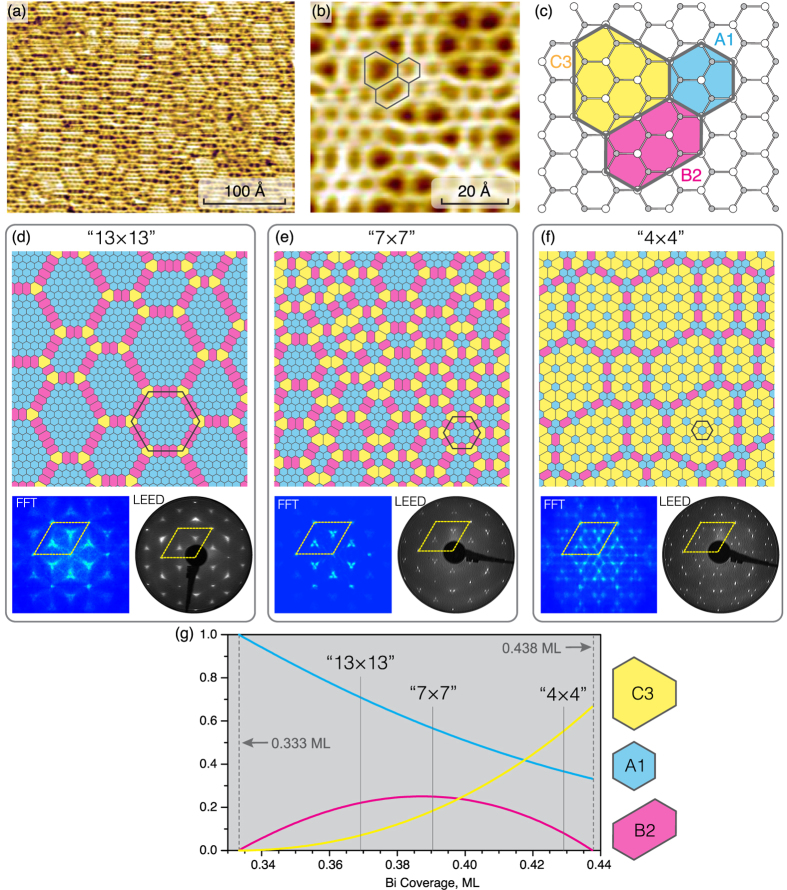
Tiling-like visualization of STM images during Tl-Bi layer formation. (**a**) STM image of the Tl-Bi surface. (**b**) Magnified fragment of the surface where three tiling elements are outlined. (**c**) Schematic diagram illustrating the shape and size of the tiling elements, A1, B2 and C3. The central panels show the simulated tiling patterns of surfaces, FFT patterns from these simulated surfaces and experimental LEED patterns from the real surfaces at these growth stages for quasi-periodic (**d**) “13 × 13”, (**e**) “7 × 7” and (**f** ) “4 × 4” (Tl, Bi)/Si(111) structures. Wigner-Seitz unit cells for the correspondent ideal periodic superstructures are outlined. (**g**) Evolution of the fractions of A1, B2 and C3 tiling elements (blue, magenta and yellow curves, respectively) during Tl-Bi compound formation shown as a function of Bi coverage.

Formation of the Tl-Bi 2D compounds was conducted under ultra-high vacuum conditions by depositing Bi onto the Tl/Si(111)1 × 1 surface. The Tl/Si(111) represents essentially a bulk-truncated Si(111) surface capped by single-monolayer Tl film^26^,^27^,^28^. One monolayer (ML) equals 7.8 × 10^14^ cm^−2^, the density of the topmost Si atoms in an ideal Si(111) plane. The typical growth temperatures were from room temperature (RT) to ~150 °C. The main growth regularities were qualitatively the same but ordering of the surface structures increases with temperature. Large-scale scanning tunneling microscopy (STM) observations reveal that upon Bi deposition Tl-Bi compound domains having a brighter STM contrast and basic 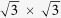 periodicity appear and grow in size with Bi dosing. High-resolution STM images in Fig. 1a,b illustrate a typical STM appearance of the Tl-Bi compound area. One can see that the image can be visualized as a tiling which superstructure is formed by three hexagonal elements, namely, A1 element having a shape of a regular hexagon with a side equal *a* = 3.84 Å, the lattice period of Si(111)1 × 1 surface, B2 element having a shape of elongated hexagon with two sides equal to 2*a* and four sides equal to *a* and C3 element having a shape of truncated triangle with three sides equal to 2*a* and three sides equal to *a* (see STM image in Fig. 1b and schematic in Fig. 1c). Remarkably, this principal arrangement of the imaging Tl-Bi surfaces preserves during structural transformations and only the relative fractions of the A1, B2 and C3 elements changes. Matching these ratios to the deposited Bi coverage it was found that each A1 element contains plausibly one Bi atom, B2 element two Bi atoms and C3 element three Bi atoms. Figure 1g illustrates how the fraction of each tiling element evolves with growing Bi coverage.

We would like to note that tiling the surface with three building blocks is very peculiar and fascinating pathway of the Tl-Bi 2D compound formation. It is in a drastic variance from a typical scenario of phase transitions at a surface during deposition of an adsorbate. Typically, nuclei of a new phase appear at initial growth stage, transforming into growing patches of a new phase with further deposition. There is always a clear structural distinction between areas occupied by an original phase and a new phase. For the Tl-Bi compound growth, quite an extraordinary situation is realized, when the quasi-periodic tiling structures evolves simultaneously all over the surface during Bi deposition via changing the relative fraction of tiling elements. Though these structures are quasi-periodic (as will be shown below), they are not actually disordered, as at each stage the surface represents an ideally-packed tiling of three basic elements without any skips or halting.

**Figure 2:**
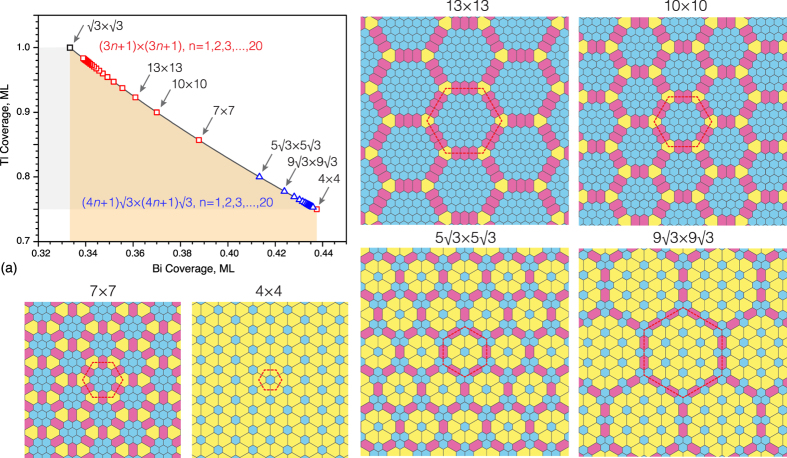
Schematic diagram illustrating idealized pathway from 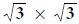-(Tl, Bi) to 4 × 4-(Tl, Bi) through the formation of a set of hypothetical periodic structures in the course of Bi adsorption. Arrangement of the tiling elements in the intermediate 13 × 13, 10 × 10, 7 × 7, 5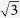 × 5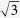, 9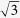 × 9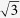 and final 4 × 4 structures are illustrated in the surrounding panels. Corresponding Wigner-Seitz unit cells of the structures are outlined in red dashed lines.

At the early growth stage, most of the surface area is occupied by 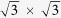 domains built of A1 elements. These domains are separated by straight domain walls built of B2 elements. The C3 elements form the domain wall triple junctions. Figure 1d shows a schematic presentation of such a surface. The A1 elements constitute there 71%, B2 elements 22%, and C3 elements 7%, Bi coverage is about 0.37 ML. Fast Fourier transform (FFT) pattern from this simulated surface is in a proper agreement with the experimental LEED pattern taken from the real (Tl, Bi)/Si(111) surface at this growth stage, i.e., both patterns display 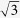 spots having triangular shape. Enlarged FFT and LEED patterns are shown in Supplementary Figure 2. Note that tiling elements with close relative ratios could be arranged also in a regular strictly-periodic structure having a 13 × 13 periodicity where the basic unit is a hexagonal domain containing 37 A1 elements surrounded by three B2 elements along each side and C3 in each corner. Corresponding Wigner-Seitz unit cell is outlined in Fig. 1d and schematic of this 13 × 13 structure is presented in Fig. 2. However, it is worth noting that this hypothetical regular structure does not appear in reality. Instead, the quasi-periodic structure develops where A1 elements build hexagonal domains most of which are irregular. The mean size of the domains corresponds to the 13 × 13 periodicity. To underline that a structure is quasi-periodic, we will write its mean periodicity in quotes, “13 × 13”.

With increasing Bi coverage, a new quasi-periodic “7 × 7” comes out at the surface (Fig. 1e) at Bi coverage of about 0.39 ML. An ideal hypothetical 7 × 7 structure has a basic unit consisting of seven A1 elements making a hexagon surrounded by a ring of alternating six B2 and six C3 elements (Fig. 2). Fourier pattern from simulated quasi-periodic “7 × 7” structure and experimental LEED pattern from the real surface show that the 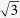 spots are split into the three individual spots which separation corresponds to the 7 × 7 periodicity (Fig. 1e). With further Bi deposition, the inter-spot separation increases indicating formation of the quasi-periodic “4 × 4” structure (Fig. 1f) at about 0.43 ML of Bi. Analysis of the ideal hypothetical structures that could occur at the surface demonstrates that in between the 7 × 7 and 4 × 4 structures the structures like 5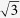 × 5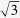 and 9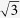 × 9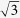 could form (see Fig. 2).

**Figure 3:**
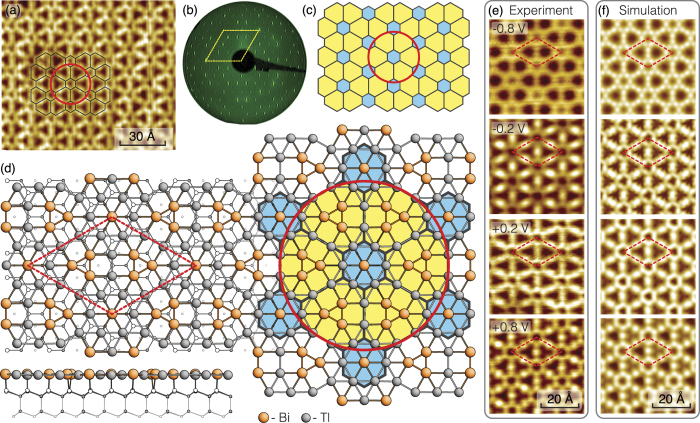
Structure of the (Tl, Bi)/Si(111)4 × 4. (**a**) STM image, (**b**) LEED pattern, (**c**) schematic diagram and (**d**) atomic arrangement (top and side views) of the (Tl, Bi)/Si(111)4 × 4 surface. Spoked-wheel-like shape of the surface basic element is indicated by red circles. Tl atoms are shown by gray circles, Bi atoms by orange circles, Si atoms by small open circles. (**e**,**f** ) show experimental and (**f** ) simulated STM images, respectively, for various sample bias voltages, −0.8, −0.2, +0.2, +0.8 V. The 4 × 4 unit cell is outlined by red dotted line.

If the growth proceeds at higher temperature of about 250 °C an almost ideal 4 × 4 periodic structure is formed, as evidenced by LEED and STM (see Fig. 3a,b and Supplementary Figure 2). The B2 elements in this structure are lacking and its basic unit consists of A1 element surrounded by six C3 elements, thus having a shape of a spoked wheel (Fig. 3c). Remarkably, this shape is not so rare for nanostructures of various size and origin. For example, nano-scale spoked wheels have been reported to be built of self-assembled triangular LaF_3_ nanoplates and Au nanoparticles^29^, short Be-encapsulated Si nanotubes^30^ and organic molecules within the void network^31^. Among them, the present atomic-size Tl-Bi spoked wheel is the smallest one, only ~2.7 nm in diameter.

In the ideal scheme, the evolution of the surface structures would start from the 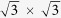-(Tl, Bi) which incorporates 1.0 ML of Tl and 0.33 ML of Bi and is built exclusively of A1 tiling elements and proceeds through a set of strictly-periodic structures (e.g., 13 × 13, 10 × 10, 7 × 7, 5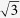 × 5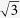, 9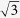 × 9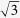 shown in Fig. 2) with various compositions of tiling elements, A1, B2 and C3, until the final 4 × 4 structure is eventually formed. In practice, upon the growth at ~150 °C the composition of the initial structure is close to that of 13 × 13 one and it is quasi-periodic. The other intermediate structures are also quasi-periodic. Solid line in Fig. 2 shows the pathway established from simulation of 150 randomly generated quasi-periodic tiling structures.

It is worth noting that, when the formation of “4 × 4”-(Tl, Bi) or 4 × 4-(Tl, Bi) structures has been completed, further Bi deposition results in the appearance of the domains of *β*-Bi/Si(111)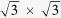 phase which grow in size until occupying eventually the whole surface at 1.0 ML Bi coverage. Remind that this reconstruction has a milk-stool structure built of Bi trimers^32^. Formation of the *β*-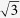-Bi phase was proved by STM and angle-resolved photoelectron spectroscopy (ARPES) observations. Simultaneously, appearance of the split spots characteristic of bulk Tl islands^33^ were detected by LEED. Thus, one can conclude that adsorbing Bi atoms substitute Tl in the metal-silicon bonds.

### Atomic structure of Tl_*x*_Bi_1−*x*_ 2D compounds

Since the 4 × 4 structure is the only 2D compound that demonstrates a well-defined long-range ordering we have concentrated our efforts on elucidating its atomic structure and electronic properties. Moreover, understanding its atomic structure provides a hint for elucidating other (Tl, Bi)/Si(111) structures. Using *ab initio* random structure searching (AIRSS)^34^, we have tested several hypothetical Tl-Bi compounds having different plausible compositions (e.g., 9 Tl atoms and 6 Bi, 10 Tl and 6 Bi atoms, 15 Tl and 6 Bi atoms, 9 Tl and 7 Bi atoms, 12 Tl and 7 Bi atoms, etc. per 4 × 4 unit cell). Among all, the structure shown in Fig. 3d has the lowest formation energy. One can see that the structure adopts 12 Tl atoms and 7 Bi atoms in the 4 × 4 unit cell. Thus, the Tl-Bi 2D compound contains 0.75 ML Tl and ~0.44 ML Bi, the total metal coverage being ~1.19 ML. Within the 4 × 4 unit cell, one Bi atom occupies the on-top (T_1_) site, the other six Bi atoms form two trimers of which the one is centered in the T_4_ site and the other in the H_3_ site. This reflects occurrence of the two types of C3 elements which is dictated by the three-fold rotational symmetry of the Si(111) surface. The Bi-Bi bond length in both trimers are almost the same being equal to 3.2 Å. Thallium atoms occupy the bridge positions between the T_4_ and H_3_ sites. Remarkably, Tl and Bi atoms are confined within a single flat atomic layer. The layer is located 2.5 Å above the top Si atoms of Si(111) substrate, the height difference of atoms constituting the layer does not exceed ~0.15 Å. All bond lengths in this Tl-Bi 2D compound on Si(111) are very close to sums of corresponding empirical atomic radii^35^ (e.g., 2.7 Å and 3.5 Å for Bi-Si and Tl-Bi bonds, respectively) indicating formation of a dense layer. Close resemblance of simulated STM images to experimental ones acquired at various bias voltages (Fig. 3e,f) can serve an indication of a proper structure determination. This resemblance allows us to elucidate also that STM features seen in both polarities are associated mainly with Tl atoms (see Supplementary Figure 3). Additional confirmation of adequate structure analysis comes from the coincidence of the calculated band structure with the experimental ARPES spectra which will be discussed later.

With the knowledge on atomic structure of the 4 × 4-(Tl, Bi) compound, one can easily evaluate atomic structure of the tiling elements, A1, B2 and C3, that, in turn, allows to establish their Tl-Bi composition, as well as composition of all hypothetical ideal and quasi-periodic real structures developing during Tl-Bi compound formation. These data are summarized in the Tables 1 and 2. In particular, the compound composition can be expressed in the form of Tl_*x*_Bi_1−*x*_ and one can see that, for example, the 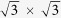-(Tl, Bi), 13 × 13-(Tl, Bi) and 4 × 4-(Tl, Bi) structures can be denoted as Tl_0.750_Bi_0.250_, Tl_0.731_Bi_0.269_ and Tl_0.632_Bi_0.368_, respectively.

### Archimedean tiling representation

Now, when atomic arrangement of all structures is defined, let us use traditional approach to describe Tl_*x*_Bi_1−*x*_ 2D compounds based on the tiling theory. One can see that Bi and Tl atoms assemble into the patterns which correspond to some of 11 Archimedean tilings first introduced by Kepler in 1619^36^. Remind that Archimedean tilings are periodic arrangements of regular polygons laid edge-to-edge in a plain. Their principal feature is that only one kind of vertex must exist, namely, where the corners of the polygons meet at a point, any given corner must always meet the same combination of corners from other polygons^37^,^38^.

**Figure 4:**
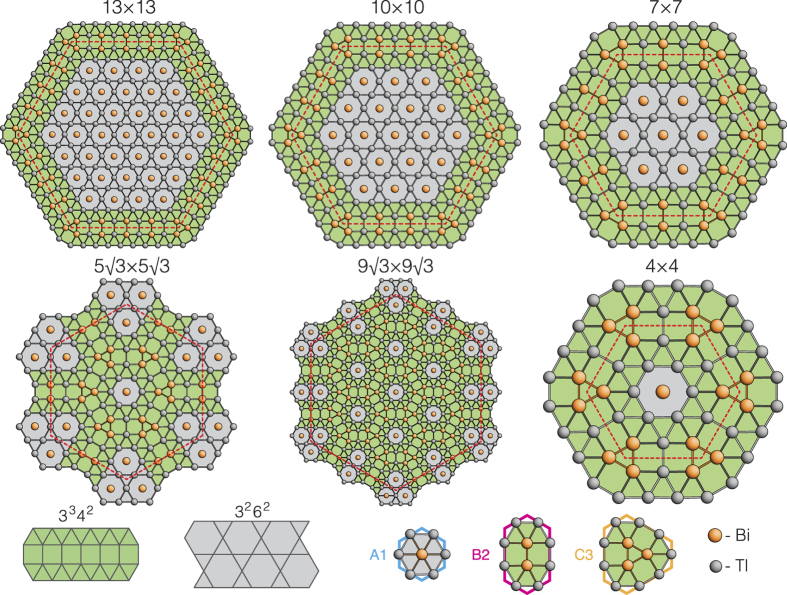
Archimedean tiling of the ideal structures, counterparts of quasi-periodic 2D Tl_*x*_Bi_1−*x*_ compounds which develop on the surface during Bi deposition. In the low right panel arrangement of the A1, B2 and C3 tiling elements are illustrated.

As one can see in Fig. 4, the idealized Tl-Bi atomic structures occurring at different formation stages (i.e., 13 × 13, 10 × 10, 7 × 7, 5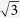 × 5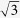, 9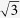 × 9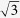 and 4 × 4) are evidently described by the two Archimedean tiling patterns. The first one (shaded in gray in Fig. 4) consists of two triangles and two hexagons alternating on each vertex (3^2^6^2^) where the vertex atoms are only Tl atoms. The second one (shaded in green in Fig. 4) is the elongated triangular tiling consisting of alternating rows of triangular and square tiles. Because each vertex is surrounded by three triangles and two squares, this leads to a (3^3^4^2^)-vertex type where the vertex atoms include both Bi and Tl atoms. If one counts all the triangles in both Archimedean tiling patterns (considering hexagons as six triangles), he will see that fraction of the triangles decreases monotonically while fraction of the squares increases with increasing Bi coverage and 4 × 4 has the lowest triangle-to-square ratio value (see Supplementary Figure 4a). This implies a less dense packing of surface atoms. Remarkably, the total metal (Tl + Bi) coverage decreases from 1.33 ML in 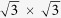-(Tl, Bi) to 1.19 ML in 4 × 4-(Tl, Bi) with increasing Bi coverage (see Supplementary Figure 4b) which looks strange taking into account that covalent radius of Bi is smaller than that of Tl. Such an unexpected behaviour might be attributed to the specific Archimedean tiling encoding of the Tl-Bi system and due to the peculiar Tl-Bi atomic coordination. Consequently, the formation energy of these structures per 1 × 1 unit cell was estimated to constitute −901 meV, −902 meV, −907 meV, −917 meV, −922 meV and −929 meV for 13 × 13, 10 × 10, 7 × 7, 5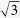 × 5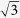, 9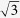 × 9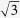 and 4 × 4 structures, respectively. Thus, the 4 × 4 is the lowest-energy structure, though its energy difference from other structures is rather small.

### Electronic band structure of Tl_*x*_Bi_1−*x*_ 2D compounds

At the early stages of Tl_*x*_Bi_1−*x*_ compound formation (i.e., when the “13 × 13” structure develops), the electron band structure is controlled mainly by the local 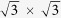-(Tl, Bi) domains. Both the experimental ARPES spectra from this surface and the band structure calculated for an ideal 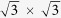-(Tl, Bi) surface demonstrate a similar metallic band (see Supplementary Figure 5). This band is spin-split and its splitting near the Fermi level is maximal in the 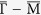 direction with the momentum splitting Δ*k*_||_ = 0.059 Å^−1^ and energy splitting Δ*E*_F_ = 222 meV.

**Figure 5:**
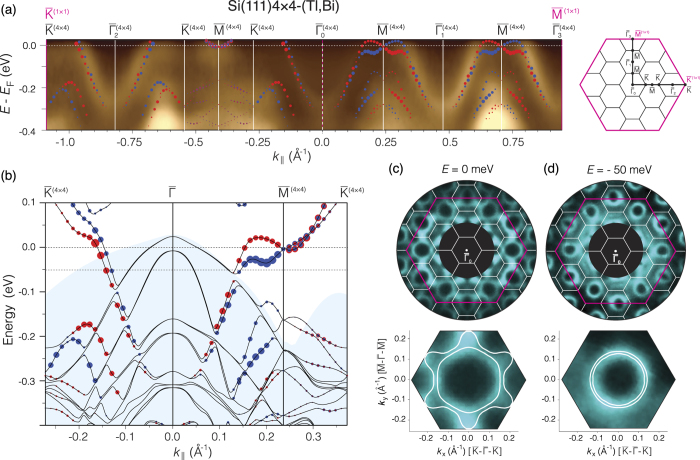
Electron band structure of the (Tl, Bi)/Si(111)4 × 4 phase. (**a**) ARPES spectrum measured in the 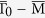 and 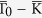 directions of the 1 × 1 SBZ, thus crossing several 4 × 4 SBZs (as indicated in the SBZ schematic diagram in the right panel where the 1 × 1 SBZ is outlined by magenta lines and the 4 × 4 SBZs by black lines). The most principal features of the calculated band structure are superposed on ARPES spectrum. (**b**) Calculated band structure in detail. The bands with opposite spin orientation is highlighted by blue and red circles. The size of the circles corresponds to the strength of the surface character summed over all orbitals at a particular *k*_||_ value. Shaded region indicates projected bulk bands. Constant energy maps taken (**c**) at the Fermi level and (**d**) 50 meV below the Fermi level. Upper panel: ARPES maps measured within the 1 × 1 SBZ. Lower panel: calculated maps (white lines) within the 4 × 4 SBZ superposed with the corresponding experimental maps cut from the maps in the upper panel. (For the enlarged constant energy maps with a detailed spin texture see Supplementary Figure 6).

When the ordered 4 × 4-(Tl, Bi) phase is formed, the surface Brillouin zone (SBZ) decreases considerably in size and electron band structure undergoes certain changes. Figure 5 summarizes the ARPES data and results of calculations on the electron band structure of the 4 × 4-(Tl, Bi) 2D compound. One can see that most distinctive feature of the band structure is the metallic surface-state band which runs upward from 0.32 eV below Fermi level at the center of the 4 × 4 SBZ (Fig. 5a,b). A simple parabolic dispersion with effective mass m^*^/*m*_0_ ≈ 0.3 fits well the band. In the ARPES constant-energy maps this band appears as a contour around 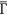 point in each 4 × 4 SBZs (Fig. 5c,d). At 50 meV below the Fermi level they show up as separate loops (Fig. 5d) but at *E*_F_ the contours are connected to their neighbors in adjacent SBZs across 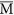 points by the “necks” (Fig. 5c). Those “necks” can be recognized in the ARPES spectrum as smudges around 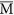 points. Due to insufficient resolution (note that 4 × 4 SBZ is relatively small), ARPES does not provide a clear visualization of the spectral features in the vicinity of the 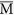 points. Fortunately, DFT calculations provides a more detailed picture. In particular, one can see that when moving from 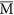 towards 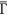 the band has a shape very similar to that reported for the spin-split band for Bi/Si(111)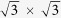 surface reconstruction^16^,^17^. Both bands display Rashba-type spin splitting, but for the 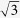-Bi the band crossing point is ~0.6 eV below *E*_F_, while for the present 4 × 4-(Tl, Bi) it is located almost at the Fermi level. The characteristic parameters quantifying the strength of the splitting are the momentum offset *k*_0_, the Rashba energy 
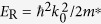
 and Rashba parameter *α*_R_ = *ħ*^2^*k*_0_/m^*^. For the 4 × 4-(Tl, Bi) surface-state band in the vicinity of the 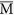 point, *k*_0_ = 0.052 Å^−1^, *E*_R_ = 26.2 meV and *α*_R_ = 1.50 eV Å (the latter value is remarkably close to *α*_R_ = 1.37 eV ⋅ Å for 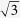-Bi^16^). In the calculated constant-energy maps (see Fig. 5c,d and detailed spin texture in Supplementary Figure 6), the split band is seen as two contours centered in the 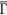 point. At the Fermi level (Fig. 5c), the inner contour appears as almost circular smoothed hexagon which shape is typical for surface-state bands on the surfaces having *C*_3*v*_ symmetry. The outer contour has a more exotic camomile-like shape, which “petals” directed towards 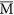 points show up as “necks” in the experimental ARPES Fermi map. The inner contour (see Supplementary Figure 6) demonstrate the clockwise helicity for in-plane spin and alternating-sign out-of-plane spin component obeying the *C*_3*v*_ symmetry which is characteristic of the Rashba-split metallic states at adsorbate-modified silicon surfaces^39^. It is remarkable that the outer contour in spite of its warped “camomile-like” shape show well defined counterclockwise spin helicity with small radial spin component even in the “petals” sections. When going down from Fermi level (e.g., at 50 meV below *E*_F_) the contours transform into the two simple concentric circular loops in agreement with the ARPES data (Fig. 5d) keeping their spin helicity.

## Conclusions

In conclusion, we synthesised and characterized two-dimensional one-atomic-layer-thick Tl_*x*_Bi_1−*x*_ compounds which display a very unusual arrangement. In contrast to the typical two-dimensional crystals which structure is built by copying a single unit cell, the found Tl_*x*_Bi_1−*x*_ compounds are composed by a set of tiles. These tiles are ideally packed tile-to-tile without any skips or halting to form highly-ordered albeit quasi-periodic structures. Structural transformations of the Tl_*x*_Bi_1−*x*_ compounds were revealed to be caused by Tl substitution for Bi and to show up as changing relative fractions of basic tiling elements. Atomic arrangement of the tiling elements and the resultant Tl_*x*_Bi_1−*x*_ compounds are fully described in detail using LEED, STM and AIRSS. Among the quasi-periodic compounds, the two, Tl_0.75_Bi_0.25_ and Tl_0.632_Bi_0.368_ are periodic with 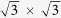 and 4 × 4 periodicity, respectively. ARPES and DFT results demonstrate that both compounds have well-defined spin-split metallic surface states with Rashba-type spin helicity. The shown example demonstrates possibility for the formation of the exotic low-dimensional materials via unusual concerted growth mechanisms.

## Methods

### Sample preparation and characterization

All experiments, including Tl_*x*_Bi_1−*x*_ 2D compounds growth and their characterization with LEED, STM and ARPES, were performed in the same three-chamber ultra-high-vacuum (UHV) Omicron MULTIPROBE system with a base pressure better than ~2.0 × 10^−10^ Torr. The UHV conditions were preserved throughout the whole experimental cycle with a working pressure being always below ~5.0 × 10^−10^ Torr. Atomically-clean Si(111)7 × 7 surfaces were prepared *in situ* by flashing to 1280 °C after the chemically pre-cleanned samples were first outgassed at 600 °C for several hours. Pristine Tl/Si(111)1 × 1 surface was prepared by depositing 1.0 ML of Tl from the heated tantalum tube onto Si(111)7 × 7 surface held at ~300 °C. Tl_*x*_Bi_1−*x*_ 2D compounds were grown by depositing Bi from the heated BN crucible onto the Tl/Si(111) surface held at a desired temperature ranging from RT to 250 °C.

STM images were acquired using Omicron variable-temperature STM-XA microscope operating in a constant-current mode. Mechanically cut Pt-Ir tips were used as STM probes after annealing in vacuum.

ARPES measurements were conducted in the ultrahigh vacuum chamber Omicron MULTIPROBE using VG Scienta R3000 electron analyzer and high-flux He discharge lamp (*hν* = 21.2 eV) with toroidal-grating monochromator as a light source.

### DFT

We searched for hypothetical TlBi compounds using recently developed AIRSS method^34^. The searching structures had been optimized based on density functional theory (DFT) with spin-orbit coupling effect included and were done using the plane-wave-based Vienna ab initio simulation package (VASP)^40^. The interactions between the ions and valence electrons were treated by the projector augmented-wave (PAW) method^41^,^42^. We used the local spin-density approximation^43^ for the exchange-correlation energy functional. For electronic band structure calculations for 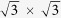-(Tl, Bi) and 4 × 4-(Tl, Bi) surface phases geometries were simulated by a repeating slab of ten Si bilayers with TBi layer lying on it and a vacuum region of ~16 Å. Si atoms in the five bottom bilayers were fixed at their bulk positions, Tl-Bi layer and top five Si bilayers were allow to fully relax, and dangling bonds on the bottom surface were saturated by hydrogen atoms. The geometry optimization is performed until the residual forces were smaller than 10 meV/Å. The kinetic cutoff energy was 250 eV, and a Monkhorst-Pack 5 × 5 × 1 and 3 × 3 × 1 *k*-point meshes were used to sample the surface Brillouin zone for 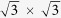-(Tl, Bi) and 4 × 4-(Tl, Bi) structures, respectively.

## Additional Information

**How to cite this article**: Gruznev, D. V. *et al.* Synthesis of two-dimensional Tl*_x_*Bi_1–x_ compounds and Archimedean encoding of their atomic structure. *Sci. Rep.* **6**, 19446; doi: 10.1038/srep19446 (2016).

## Supplementary Material

Supplementary Information

## Figures and Tables

**Table 1:** Tl-Bi composition of tiling elements, A1, B2 and C3.

Element	Area, 1 × 1 units	N of atoms		Coverage, ML		Elements	
		Tl	Bi	Θ_Tl_	Θ_Bi_	Δ	□
A1	3	3	1	1.000	0.333	8	0
B2	5	4	2	0.800	0.400	8	2
C3	6.5	4.5	3	0.692	0.462	9	3

**Table 2:** Composition of the idealized periodic tiling structures.

Unit cell	Number of elements (%)			N of atoms		Coverage, ML		Tl_*x*_Bi_1−*x*_
	A1	B2	C3	Tl	Bi	Θ_Tl_	Θ_Bi_	
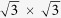	1 (100%)	0 (0%)	0 (0%)	3	1	1.000	0.333	Tl_0.750_Bi_0.250_
13 × 13	37 (77.0%)	9 (18.8%)	2 (4.2%)	156	61	0.923	0.361	Tl_0.731_Bi_0.269_
10 × 10	19 (70.4%)	6 (22.2%)	2 (7.4%)	90	37	0.900	0.370	Tl_0.709_Bi_0.291_
7 × 7	7 (58.3%)	3 (25.0%)	2 (16.7%)	42	19	0.857	0.388	Tl_0.689_Bi_0.311_
5 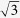 × 5 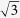	7 (43.8%)	3 (18.8%)	6 (37.5%)	60	31	0.800	0.413	Tl_0.659_Bi_0.341_
9 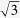 ×9 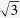	19 (38.9%)	6 (12.2%)	24 (49.0%)	189	103	0.778	0.424	Tl_0.647_Bi_0.353_
4 × 4	1 (33.3%)	0 (0%)	2 (66.7%)	12	7	0.750	0.438	Tl_0.632_Bi_0.368_

Numbers of tiling elements and atoms are given per corresponding unit cell. The structures are listed in the order of increasing Bi coverage.
