# Supplementary material for: Synthesis of two-dimensional TlxBi1−x compounds and Archimedean encoding of their atomic structure
Source: Sci Rep. 2016 Jan 19;6:19446. doi: 10.1038/srep19446 (PMC4726083; doi:10.1038/srep19446)
Supplement: Supplementary Information [file srep19446-s1.pdf]

Supplementary Information for Synthesis of two-dimensional Tl_xBi_{1-x} compounds and Archimedean encoding of their atomic structure

Dimitry V. Gruznev, Leonid V. Bondarenko, Andrey V. Matetskiy, Alexey N. Mihalyuk, Alexandra Y. Tupchaya, Oleg A. Utas, Sergey V. Eremeev, Cheng-Rong Hsing, Jyh-Pin Chou, Ching-Ming Wei, Andrey V. Zotov & Alexander A. Saranin

1. Enlarged FFT and LEED patterns

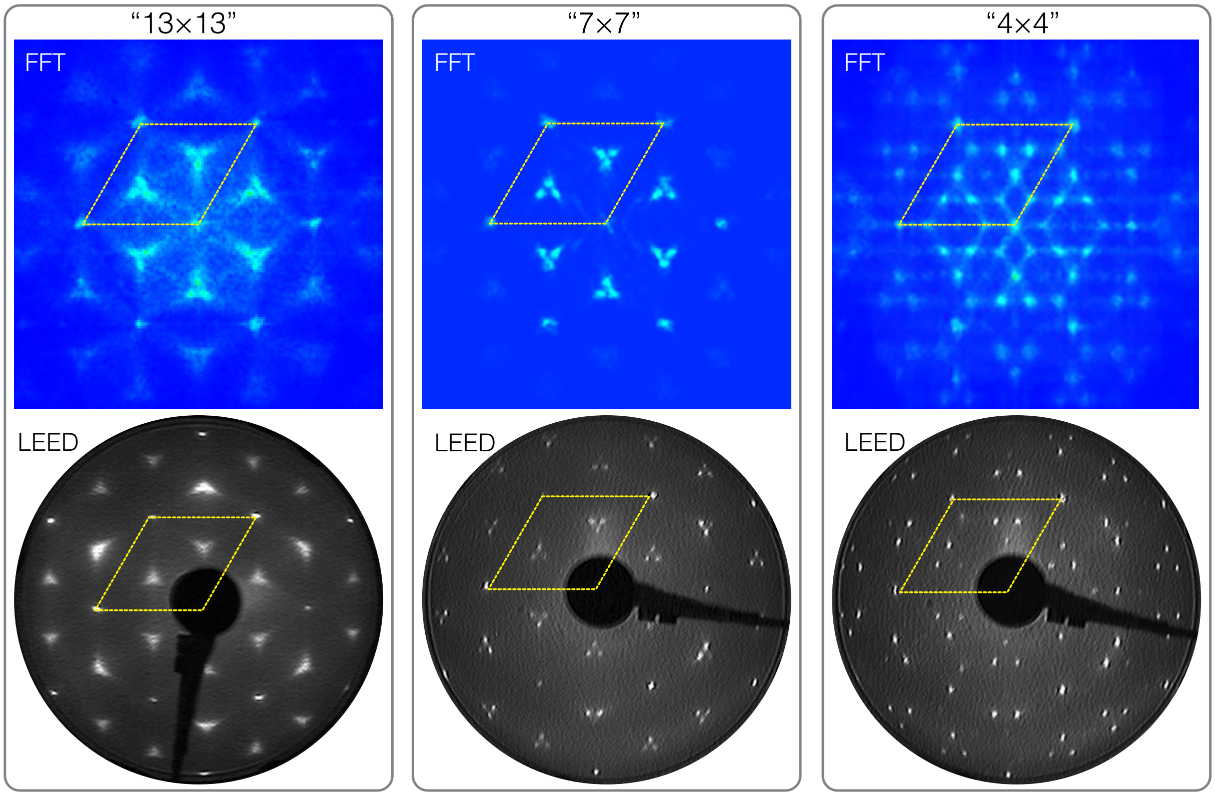

Figure 1: Enlarged FFT patterns from the simulated structures and experimental LEED patterns from the real surfaces for quasi-periodic "13×13", "7×7" and "4×4" (Tl, Bi)/Si(111) structures.

2. Si(111)4×4-(Tl, Bi): large-scale view

Large-scale STM images and sharp 4×4 LEED patterns with low background and absence of any other reflections (Fig. 2) indicate that the Si(111)4×4-(Tl, Bi) surface demonstrates exclusively high ordering and homogeneity over extended areas.

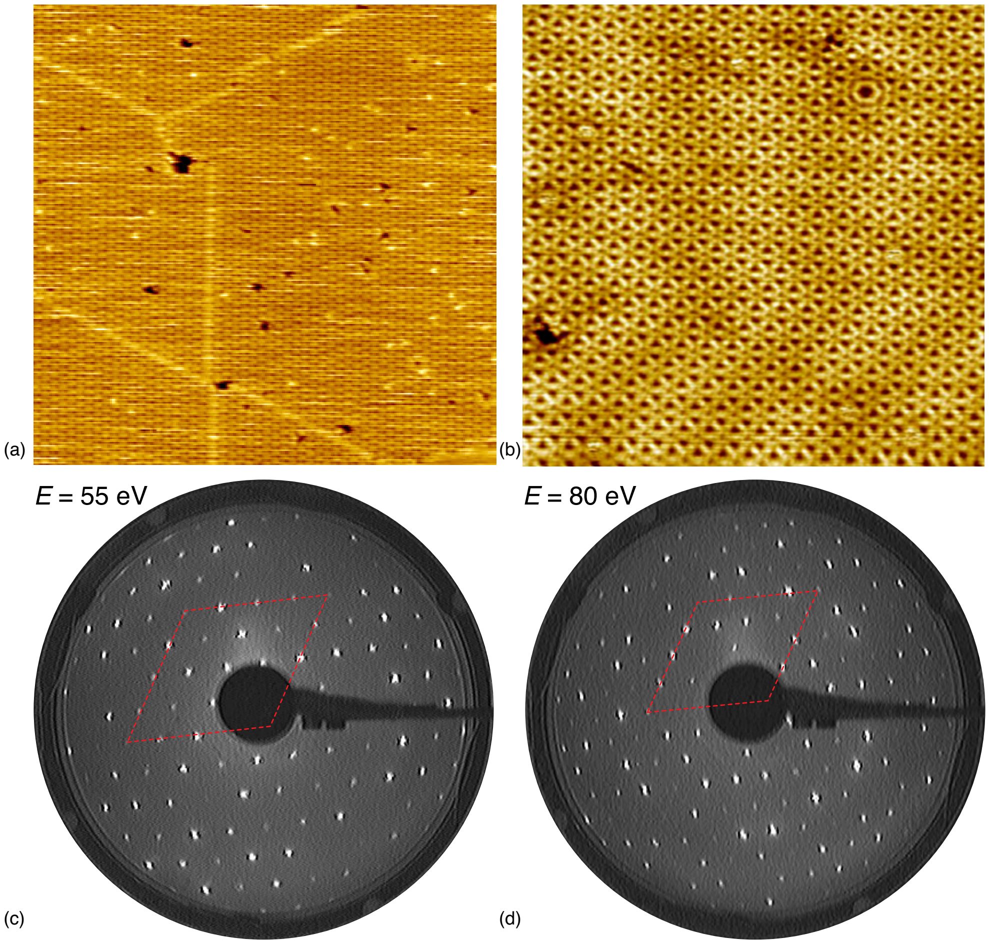

Figure 2: (a) 750×750 Å^2 and (b) 300×300 Å^2 STM images and LEED patterns taken at primary energy beam E_p of (c) 55 eV and (d) 80 eV from Si(111)4×4-(Tl, Bi) surface. The 1×1 unit cells in the LEED patterns are outlined by red frames to guide an eye.

3. Conformity of 4×4-(Tl, Bi) atomic structure and STM

One can see from Fig 3 showing superposition of the 4×4-(Tl, Bi) atomic structural model and corresponding STM image that STM protrusions coincide with the location of Tl atoms, while deep triangular depressions are associated with Bi trimers and shallow round depressions with Bi adatoms.

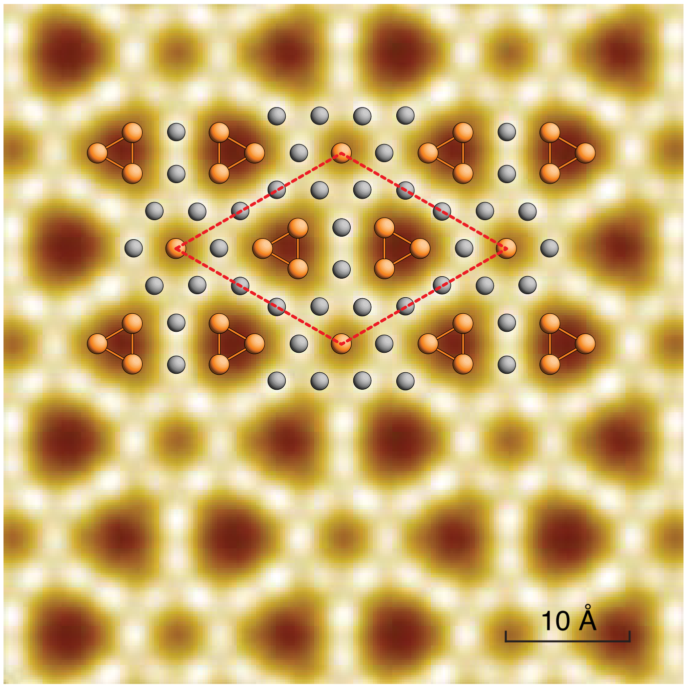

Figure 3: Structural model of the 4×4-(Tl, Bi) 2D compound superposed on the STM image. Tl atoms are shown by gray circles, Bi atoms by orange circles. 4×4 unit cell is outlined with dashed red frame.

4. Evolution of Archimedean tiling features during Tl_xBi_{1-x} 2D compound formation

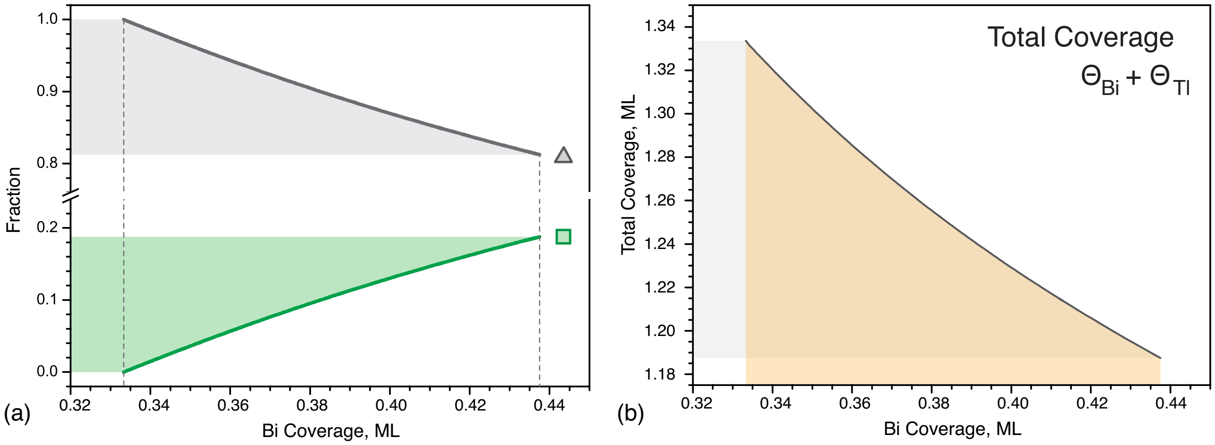

Figure 4: (a) Fraction of triangles (gray curve) and squares (green curve) in the tiling patterns versus Bi coverage. (b) Total metal coverage (Tl + Bi) versus Bi coverage.

5. Electronic band structure of the "13×13"-(Tl, Bi) compound

Surface-state band of the quasi-periodic "13×13"-(Tl, Bi) compound demonstrates a clear spin splitting predicted by the calculation results for an idealized $\sqrt{3}\times\sqrt{3}$-(Tl, Bi) surface (Fig. 5).

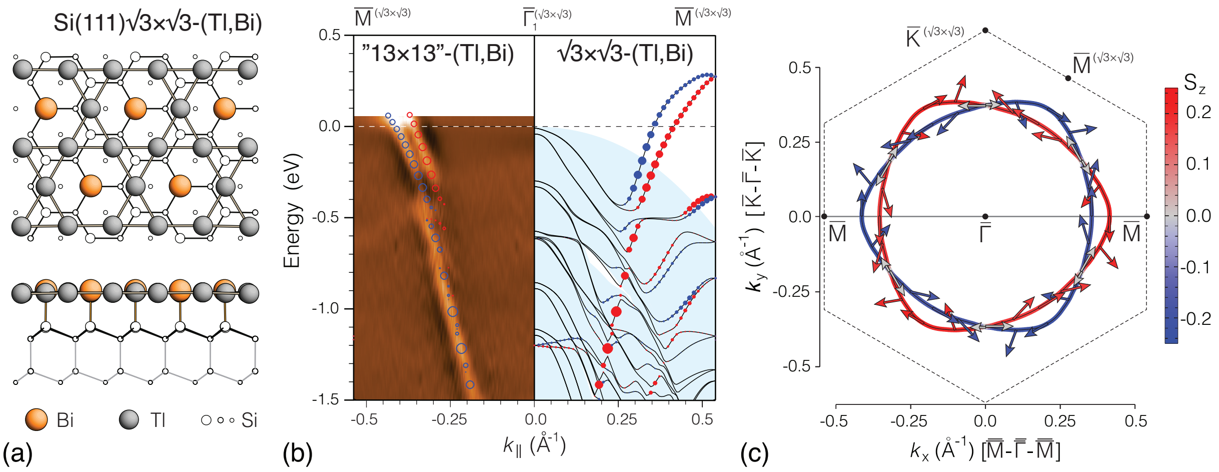

Figure 5: Basic atomic arrangement and electron band structure of the Tl_xBi_{1-x} layer on the stage of "13×13" superstructure formation. (a) Structural model of the $\sqrt{3}\times\sqrt{3}$-(Tl, Bi) domain. Tl atoms are shown by gray circles, Bi atoms by orange circles, Si atoms by small white circles. (b) Comparison of the experimental ARPES specrum from the "13×13"-(Tl, Bi) layer (left panel) with the calculated band structure for an ideal $\sqrt{3}\times\sqrt{3}$-(Tl, Bi) phase. The bands with opposite spin orientation is highlighted by blue and red circles. The size of the circles corresponds to the strength of the surface character summed over all orbitals at a particular $k_{\|}$ value. Shaded region indicates projected bulk bands. (c) Calculated Fermi map for the $\sqrt{3}\times\sqrt{3}$-(Tl, Bi).

6. Spin texture of the 4×4-(Tl, Bi) compound

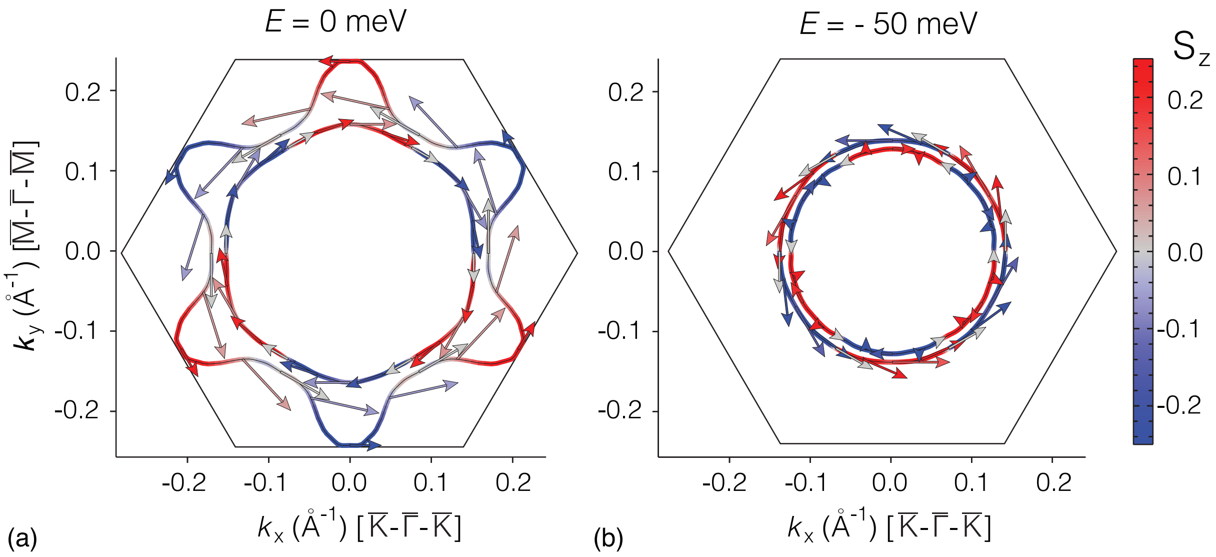

Figure 6: Calculated constant energy maps (a) at the Fermi level and (b) 50 meV below the Fermi level. The out-of-plane spin component is indicated by the colour with red and blue corresponding to the upward and downward directions, respectively. Light gray colour indicates fully in-plane spin alignment. Arrows adjacent to the calculated contours and their length denote the in-plane spin component.
